# Hepatoprotective effect of *Solanum surattense* leaf extract against chemical- induced oxidative and apoptotic injury in rats

**DOI:** 10.1186/s12906-019-2553-1

**Published:** 2019-07-03

**Authors:** Mohammad K. Parvez, Mohammed S. Al-Dosari, Ahmed H. Arbab, Perwez Alam, Mansour S. Alsaid, Azmat A. Khan

**Affiliations:** 1 Department of Pharmacognosy, College of Pharmacy, King Saud University, Riyadh, Saudi Arabia; 2 Department of Pharmacognosy, Faculty of Pharmacy, University of Khartoum, Khartoum, Sudan; 3 Medicinal, Aromatic and Poisonous Plants Research Center, College of Pharmacy, King Saud University, Riyadh, Saudi Arabia; 4 Department of Pharmaceutical Chemistry, College of Pharmacy, King Saud University, Riyadh, Saudi Arabia

**Keywords:** *Solanum surattense*, Hepatoprotection, Oxidative stress, Apoptosis, DCFH, CCl_4_, β-sitosterol

## Abstract

**Background:**

Of over 35 Saudi plants traditionally used to treat liver disorders, majority still lack scientific validations. We therefore, evaluated the anti-oxidative, anti-apoptotic and hepatoprotective potential of *Solanum surattense* leaves total ethanol-extract (SSEE).

**Methods:**

The cytoprotective (4,5-dimethylthiazol-2-yl-2,5-diphenyltetrazolium bromide/ MTT assay) and anti-apoptotic (caspase-3/7) potential of SSEE (25–200 μg/mL) were assessed in cultured HepG2 cells against dichlorofluorescein (DCFH)-induced toxicity. The hepatoprotective salutation of SSEE (100 and 200 mg/kg.bw/day) in carbon tetrachloride (CCl_4_)-intoxicated rats was evaluated by serum biochemistry and histopathology. The anti-oxidative activity of SSEE (31.25–500 μg/mL) was tested by 1,1-diphenyl-2-picrylhydrazyl (DPPH) radical-scavenging and linoleic acid bleaching assays. Also, SSEE was subjected to qualitative phytochemical analysis, and standardized by validated high-performance liquid chromatography (HPTLC).

**Results:**

SSEE at doses 50, 100 and 200 μg/mL showed HepG2 cell proliferative and protective potential by about 61.0, 67.2 and 95%, respectively through inhibition of caspase-3/7 against DCFH-toxicity. In CCl_4_-injured rats, SSEE (200 mg/kg) significantly (*P* < 0.001) normalized serum transaminases, alkaline phosphatase, bilirubin, cholesterol, triglycerides, and total protein, including tissue malondialdehyde and nonprotein sulfhydryls levels, supported by the liver histopathology. SSEE further showed strong in vitro anti-oxidative and anti-lipid peroxidative activities, evidenced by the presence of alkaloids, flavonoids, tannins, sterols and saponins. Identification of β-sitosterol (3.46 μg/mg) strongly supported the anti-oxidative and hepatoprotective salutation of SSEE.

**Conclusion:**

Our findings suggest the therapeutic potential of *S. surattense* against chemical-induced oxidative stress and liver damage. However, isolation of the active principles and elucidation of mechanism of action remain to be addressed.

## Background

Liver links the digestive tract and general circulation, and plays a central role in nutrient metabolism, synthesis of functional proteins and detoxification of drugs and chemicals [1, 2]. Liver is subjected to various diseases which may be metabolic, toxin/chemical-induced tissue inflammation or viral hepatitis B/C-associated cirrhosis and carcinomas. Chronic liver disease is the fifth most common cause of mortality, worldwide where the casualty rate is increasing despite of advances in therapeutics [2]. Therefore, there is an essential need to search for new anti-hepatitis and hepatoprotective agents [3]. Notably, despite the significant popularity of several mono and poly-herbal products for liver diseases [4], only few, including silymarin are approved [5].

In Saudi Arabia, about 100 species of medicinal plants have been documented, and of these > 35 plants are used in traditional folk medicine for the treatment of liver disorders [6, 7]. However, most of these herbal preparations are not subjected to sustained scientific evaluation. Therefore, based on known Saudi traditional use and available literature, we recently screened over seventy plants, notably *Acacia mellifera*, *Aerva javanica*, *Atriplex subrecta* and *Solanum surattense* for their cytotoxic, growth stimulating, and anti-hepatitis B activities in cultured liver cells [8, 9]. Of these, further investigation of *A. mellifera* [9], *A. javanica* [10] and *A. subrecta* [11] showed strong anti-oxidative and hepatoproective efficacy in cultured liver cells as well as in rodents.

*S. surattense* Burm F. (Solanaceae), a perennial herbaceous weed that grows in the south of Saudi Arabia, Farasan islands and India. It is traditionally used for treatment of fever, asthma, cough, toothache, sexual diseases and to promote female fertility [7, 12]. Moreover, ample of biological screenings of *S. surattense* revealed that it had a significant anti-fungal [13], anti-plasmodial [14], anti-hyperlipidemic [15] and anti-diabetic [16] efficacies. In addition, while *S. surattense* seeds aqueous extract has been shown to deplete the oxidative stress of cauda epididymal spermatozoa [17], its **e**thanol extract is recently shown to have diuretic activity [18] in albino rats. A rare 16β-H steroidal alkaloid saponin and six known saponins [19], including two new steroidal alkaloids, solanoside A1 and solanoside B2 have been isolated from *S. surattense* [20]. With this back ground information and in line with our recent findings [8–11], the present study extends further investigation of *S. surattense* for the anti-oxidative, anti-apoptotic and hepatoprotective potential.

## Methods

### Plant material collection and preparation of total ethanol-extract

*S. surattense* was collected from Jazan, Saudi Arabia and authenticated by an expert taxonomist Prof. Mohammad Yusuf at the College of Pharmacy, King Saud University, Riyadh, and a voucher specimen (no. 16386) was submitted at the college herbarium. The plant leaves were washed, air-dried, powdered (300 g), soaked in 70% ethanol (Merck, Germany) for two days at room temperature (RT), and filtered through Whatmann filter paper No.1 (Whatmann, USA). The process was repeated twice in ethanol and the extracts were evaporated under reduced pressure at 4 °C (Rotary Evaporator; Buchi, Switzerland). The obtained semi-solid *S. surattense* total ethanol-extract (SSEE; 31.5 g) was stored at -20 °C until used.

### Drugs and compounds

2,7-Dichlorofluorescein (DCFH; Sigma, USA) and carbon tetrachloride (CCl_4_; Merc, Germany) was used to induce in vitro and in vivo hepatocytotoxicity, respectively. Silymarin (Sigma, USA) was used as standard hepatoprotective drug. Ascorbic acid (Sigma, USA), gallic acid (Fluka, USA) and β-sitosterol (Sigma, USA) were used as standard antioxidant agents.

### Human liver cell culture

Human hepatoblastoma cells, HepG2 (Cat# ATCC® HB-8065) were procured (American Type Culture Collection, VA, USA) and grown in T75 culture flasks (Corning, USA) at 37 °C with 5% CO_2_ supply. The complete culture medium, RPMI-1640 (Gibco, UAS) contained 10% bovine serum (Gibco, USA) and 1x penicillin-streptomycin mix (HyClone Laboratories, USA).

### In vitro hepatoprotective and apoptotic signaling assays

HepG2 cells were seeded (0.5 × 10^5^ cells/well, in triplicate) in a 96-well flat-bottom plate and grown over night. DCFH (IC_50_: 100 μg/mL) was used as a chemical inducer of hepatocyte toxicity [9–11]. Four different doses of SSEE (25, 50, 100 and 200 μg/mL) and DCFH were prepared using dimethyl sulfoxide (DMSO; > 0.1%, final) and complete RPMI media. For hepatoprotection assay, the cultures were treated with DCFH (100 μg/mL) plus a dose of SSEE, including untreated and DCFH alone-treated controls. The cells were incubated for 48 h, following MTT assay (TACS MTT Cell Proliferation Assay Kit, Trevigen, USA) as per the manufacturer’s instruction, and the optical density (OD) was measured (Microplate Reader ELx800; BioTek, USA).

For anti-apoptotic assay, caspase-3/7 activation was measured (Apo-ONE-homogenous caspase‑3/7 Assay Kit; Promega, USA) as per the supplied manual. Briefly, HepG2 toxicity was induced with DCFH as above, and treated with the four doses of SSEE for 48 h. Caspase-3/7 reagent was added (100 μL/well), incubated in dark at RT for 5 h, and the OD was measured.

For both the assays, cell survival was determined using the equation: *[(OD*_*s*_*-OD*_*b*_*)/(OD*_*c*_*-OD*_*b*_*)] × 100* where OD_s_, OD_b_ and OD_c_ are the optical density of sample, blank and negative control, respectively. Data were subjected to non-linear regression analysis (Excel software 10.0, Microsoft, CA, USA) and presented as % cell survival in relation to the untreated control.

### Animals, experimental design and SSEE treatment

Thirty male Wistar rats (200–220 g; 8–9 wks) obtained from the Experimental Animal Care Center (College of Pharmacy, KSU, Riyadh) were kept in polycarbonate cages in a temperature-controlled sterile chamber under 12 h dark/light cycle (25 ± 2 °C). After acclimatization, animals were randomized and divided into five groups (Group I–Group V; six each). Group I served as untreated control and orally administered with normal saline (1.0 mL). Group II, III, IV and V received CCl_4_ in liquid paraffin (1:1; 1.25 mL/kg·bw), intraperitoneally (i.p.). Groups III and IV were orally administered with SSEE at a dose of 100 and 200 mg/kg·bw, respectively for three weeks. Group V was orally administered with silymarin (10 mg/kg·bw) [21] for three weeks. All animals were cared in compliance with the guidelines of the Ethics Committee of the Experimental Animal Care Society, King Saud University, Riyadh.

### Acute oral toxicity test

Rats were divided into five groups (GI-V, six animal each), and acute oral toxicity was performed (G1: normal control; GII: 50 mg/kg.bw; GIII: 100 mg/kg.bw; GIV: 200 mg/kg.bw and GV: 500 mg/kg.bw) using the limit test as per the OECD guidelines for acute toxicity test 401 [22]. Animals were observed continuously for 1 h and then for 4 h at 30 min intervals, for any gross behavioral change and general motor activities (eg., writhing, convulsion, response to tail pinching, gnawing, pupil size, fecal output, feeding behavior, etc.), followed by an additional 72 h observation for healthy survival.

### Blood collection and liver tissue preparation

Animals were anesthetized with sodium pentobarbital (Sigma-Aldrich, Germany; 50 mg/kg.bw; i.p.) and sacrificed by cervical dislocation following blood collection (21G syringe). The liver tissues were quickly dissected, washed (1x PBS), fixed (10% neutral buffered formalin) for 72 h, and processed overnight for dehydration and paraffin impregnation using Automatic Tissue Processor (Sakura, Japan). The specimens were embedded in paraffin blocks using Embedding Station (Sakura, Japan), cut into 4 μm sections using Rotary Microtome (Leica-RM2245, Germany) and stained with H&E (Hematoxylin & Eosin).

### Estimation of liver enzymes and bilirubin

Rats serum samples were subjected to biochemical analyses for the levels of serum alanine aminotransaminase (ALT; Reflotron GPT, Roche Diagnostics GmbH, Mannheim, Germany), spartate aminotransaminase (AST; Reflotron GOT, Roche Diagnostics GmbH, Mannheim, Germany), alkaline phosphatase (ALP; Reflotron ALP, Roche Diagnostics GmbH, Mannheim, Germany), γ-glutamyl transferase (GGT; Reflotron GGT, Roche Diagnostics GmbH, Mannheim, Germany) and bilirubin (BIL; Reflotron BIL, Roche Diagnostics GmbH, Mannheim, Germany), using Reflotron Plus Analyzer (Woodley Equipment Co., Ltd., UK).

### Lipid profiling

The levels of serum triglycerides (TG; Reflotron TG, Roche Diagnostics GmbH, Mannheim, Germany), total cholesterol (TC; Reflotron Cholesterol, Roche Diagnostics GmbH, Mannheim, Germany) and high-density lipoproteins (HDL; Reflotron HDL Cholesterol, Roche Diagnostics GmbH, Mannheim, Germany) were determined using Reflotron Plus Analyzer (Woodley Equipment Co., Ltd., UK). For each sample, very low-density lipoproteins (VLDL) and low-density lipoproteins (LDL) were determined using the standard formula: *VLDL = TG/5* and *LDL = TC-(VLDL + HDL)*, respectively.

### Estimation of total protein

The rats serum total protein (TP) level were analyzed using commercial kit (Crescent Diagnostics Kit, Jeddah, Saudi Arabia), and calculated (*TP = [OD*_*sample*_*/OD*_*standard*_*] × Concentration of standard*) where OD_sample_ and OD_standard_ are the optical density of sample and standard, respectively.

### Determination of tissue malondialdehyde

The liver tissue malondialdehyde (MDA) was determined as described elsewhere [23]. Briefly, tissues were homogenized with Potter-Elvehjem Type-C Homogenizer (Thomas scientific Inc., USA) in ice-cold potassium chloride (KCl; 0.15 M). The OD (λ = 532 nm) was recorded and the MDA content (nmol/g wet tissue) was calculated with reference to a standard curve of the freshly prepared MDA solution.

### Estimation of tissue nonprotein sulfhydryls

Liver tissue nonprotein sulfhydryls (NP-SH) concentrations were measured as described elsewhere [24]. Briefly, tissues were homogenized with Potter-Elvehjem Type-C Homogenizer (Thomas scientific Inc., USA) in ice-cold ethylenediaminetetraacetic acid (EDTA; 0.02 mM) and the OD (λ = 412 nm) was measured after adding Ellman’s reagent (DTNB, 5,5’dithio-bis-2-nitrobenzoic acid; 0.02 mL).

### Microscopy and histopathology

Treated HepG2 cells were observed under an inverted microscope (Optica, × 40 and × 100) for any morphological changes at 24 and 48 h. Liver histopathology was examined under OMX1200C Light Microscope (Nikon, Japan) at magnifications of × 200 and × 400, and images were recorded with mounted digital camera.

### 1,1-diphenyl-2-picrylhydrazyl (DPPH) radical scavenging activity of SSEE

The in vitro free-radical scavenging ability of SSEE against DPPH was evaluated quantitatively as described elsewhere [25] in a 96-well microplate [10, 11]. Briefly, the triplicated dose of SSEE (31.25, 62.5, 125, 250 and 500 μg/mL) in a volume of 100 μL/well was mixed with 40 μl of DPPH (0.2 mM in methanol), including ascorbic acid (standard) and a solvent control. Following 30 min incubation at 25 °C, OD (λ = 517 nm) was read and percent radical scavenging activity was calculated [1-(*OD*_*sample*_/*OD*_*standard*_) × 100, where OD_sample_ and OD_standard_ where OD_sample_ and OD_standard_ are the optical density of sample and standard, respectively].

### β-Carotene-linoleic acid bleaching assay of SSEE

The in vitro anti-oxidant (anti-lipid peroxidation) activity was evaluated as described elsewhere [26] in a 96-well plate [10, 11]. Briefly, 0.25 mg β-carotene was first dissolved in 0.5 mL chloroform, and added to a 12.5 μg linoleic acid and 100 mg Tween-40 solution, following chloroform evaporation (Speed vacuum concentrator; Savant, Thermo Electron Co.). The resultant was immediately diluted to 25 mL with distilled water and shaken to form an emulsion. A 200 μL aliquot/well was added to SSEE (50 μL) or gallic acid (500 μg/mL), including a solvent control (all in triplicate). Following incubation at 50 °C for 2 h, OD (λ = 470 nm) was read at 30 min intervals. The antioxidant activity was estimated using two methods: by plotting a kinetic curve of sample against time, and by estimating the percent inhibition of lipid peroxidation (*[ODs*_*120*_ *– ODc*_*120*_*/ ODc*_*0*_*– ODc*_*120*_*] × 100)* where As_120_ and Ac_120_ are the optical density of the sample and control, respectively at 120 min, and OD_0_ is the optical density of the control at 0 min.

### Qualitative phytochemical screening

The preliminary in vitro qualitative phytochemical screening of SSEE for major secondary metabolites, such as alkaloids, flavonoids, anthraquinones, tannins, saponins, sterols and cardiac glycosides was performed using standard procedures [27]. Briefly, for alkaloids 3 mL solution of SSEE was dissolved in hydrochloric acid (HCl; 2%) filtered, and added to Mayer’s reagent prepared in distilled water (50 mL, final volume) where formation of a yellow precipitate was the positive test. For flavonoids, 5 mL SSEE solution was treated with drops of sodium hydroxide (NaOH; 20%) where appearance of yellow color that turned colorless after addition of diluted hydrochloric acid was the confirmation. For tannins, 0.25 mg SSEE dissolved in 10 mL water and mixed with several drops of ferric chloride (FeCl_3_; 5%) where development of a brown-green/blue-black color was the sign of positivity. For saponins, 0.5 mg SSEE dissolved in 10 mL water was agitated vigorously to form a thick persistent froth presenting a positive result.

### Standardization of SSEE by validated high-performance thin layer chromatograpy

The high-performance thin layer chromatography (HPTLC) method was developed and validated to standardize the 70% SSEE as described elsewhere [28]. Briefly, HPTLC (10 × 10 cm pre-coated silica gel F_254_ plate) was carried out using β-sitosterol as reference biomarker. Of the several mobile phases tried to get good resolution and separation of different compounds present in SSEE, hexane and ethyl acetate (4:6; v/v) was selected as the best combination. The standard along with samples was applied on the HPTLC plate (CAMAG Automatic TLC Sampler-4), developed (CAMAG Automated Developing Chamber-2), and scanned (λ = 360 nm; CAMAG TLC Scanner-3) for densitometric analysis.

### Statistical analysis

Results were expressed as mean ± S.E.M. Total variation present in a set of data was estimated by one-way analysis of variance (ANOVA) followed by Dunnet’s-test (Excel 2010; Microsoft OK, USA). *P* < 0.05 was considered significant.

## Results

### Hepatoprotective and anti-apoptotic effect of SSEE on cultured liver cells

**Fig. 1:**
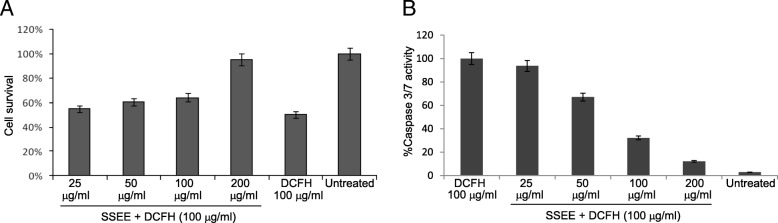
In vitro protection of cultured HepG2 cells by *S. surattense* ethanol-extract (SSEE). **a** MTT Cell proliferation assay showing attenuation of DCFH-induced oxidative damage. **b** apoptotic assay showing inhibition of DCFH-induced caspase-3/7 activation

Microscopic observation showed apoptotic effect of DCFH on HepG2 cells and morphological recovery in SSEE (50, 100 and 200 μg/mL) treated cells at 24 and 48 h (data not shown). While the MTT assay revealed attenuation of DCFH-toxicity by SSEE in a dose-dependent manner by restoring the cells proliferation by about 61.0, 67.2 and 95%, respectively (Fig. 1a), the anti-apoptotic signaling analysis showed inhibition of cellular caspase-3/7 activity by ~ 33, 68 and 88%, respectively (Fig. 1b).

### Therapeutic effects of SSEE on rat liver biochemical markers

The acute toxicity test of SSEE (limit: 500 mg/kg) showed healthy survival of the rats without any sign behavioral changes or toxicity. We therefore, utilized 100 and 200 mg/kg.bw of SSEE as experimental doses. The administration of CCl_4_ (1.25 mL/kg) drastically elevated the levels of AST, ALT, GGT, ALP, BIL, TC, TG, LDL, VLDL and MDA, and reduced HDL, TP and NP-SH contents compared to the untretaed control group, indicating liver injury while treatment with SSEE (100 and 200 mg/kg) and silymarin (10 mg/kg.bw) significantly normalized (*P* < 0.001) these parameters as compared to the CCl_4_-only group (Table 1). The estimated hepatoprotective activity of SSEE on AST, ALT, ALP, GGT, BIL, TC, TG, LDL, VLDL, MDA, HDL, TP, and NP-SH (in order) was 2, 14.2, 7%, 15, 16.2, 13, 21, 13.3, 20, 7.2, 5.7, 9.2 and 34.4% at 100 mg/kg; and 10.8, 30.7, 15.3, 30.7, 27.3, 18, 35.7, 32.7, 36.6, 43, 30.55, 31 and 36.6% at 200 mg/kg whereas that of silymarin was 53.5, 62.7, 23, 53.8, 51, 28, 27, 43.3, 26.6, 71.5, 38.1, 46.7 and 41.5%, respectively (Table 1).

**Table 1:** Therapeutic effect of SSEE against CCl_4_-induced hepatotoxicity related parameters in rats

Liver function parameters	GI (Control)	GII (CCl_4_-only)	GIII (CCl_4_+ SSEE 100 mg)	GIV (CCl_4_+ SSEE 200 mg)	GV (CCl_4_+ Silymarin 10 mg)
AST (U/L)	107.45 ± 5.31	294.83 ± 8.33***	288.50 ± 7.36 ^b^	262.50 ± 6.87^b^	136.66 ± 6.00**^b^
ALT (U/L)	28.83 ± 2.20	230.83 ± 9.62***^a^	198.16 ± 7.12*^b^	159.50 ± 9.17***^b^	85.66 ± 4.31***^b^
ALP (U/L)	321.66 ± 13.8	515.16 ± 13.7***^a^	479.00 ± 6.12* ^b^	436.33 ± 12.31**^b^	396.33 ± 7.62***^b^
GGT (U/L)	4.06 ± 0.32	12.85 ± 0.98***^a^	11.38 ± 0.49^b^	9.46 ± 0.32**^b^	5.58 ± 0.28***^b^
BIL (mg/dL)	0.54 ± 0.01	2.16 ± 0.08***^a^	1.81 ± 0.05*^b^	1.57 ± 0.06***^b^	1.06 ± 0.06***^b^
TC (mg/dL)	109.83 ± 3.9	206.00 ± 4.53***^a^	179.50 ± 6.63**^b^	168.83 ± 6.16***	147.66 ± 4.88***^b^
TG (mg/dL)	59.01 ± 2.74	151.16 ± 4.61***^a^	119.33 ± 3.8***^b^	96.81 ± 3.75***^b^	110.16 ± 5.26***^b^
HDL (mg/dL)	55.18 ± 2.40	25.25 ± 1.79***	26.53 ± 1.22^b^	36.28 ± 1.78**^b^	40.41 ± 2.97**^b^
LDL (mg/dL)	42.84 ± 3.22	149.51 ± 4.28***^a^	130.10 ± 7.50*^b^	113.18 ± 7.60**^b^	85.21 ± 5.98***^b^
VLDL (mg/dL)	11.80 ± 0.54	30.23 ± 0.92***^a^	23.86 ± 0.76***^b^	19.36 ± 0.75***^b^	22.02 ± 1.05***^b^
TP (g/L)	113.76 ± 2.8	49.11 ± 1.82***^a^	53.88 ± 2.81^b^	71.452.64***^b^	91.81 ± 4.08***^b^
MDA (nmol/g)	0.50 ± 0.02	4.82 ± 0.29***^a^	4.47 ± 0.23^b^	2.75 ± 0.1***^b^	1.37 ± 0.16***^b^
NP-SH (nmol/g)	7.39 ± 0.53	3.86 ± 0.44***^a^	5.79 ± 0.35**^b^	5.93 ± 0.31**^b^	6.52 ± 0.31***^b^

*AST* spartate aminotransaminase, *ALT* alanine aminotransaminase, *ALP* alkaline phosphatase, *GGT* γ-glutamyl transferase, *BIL* bilirubin, *TC* triglycerides, *HDL* high-density lipoproteins, *LDL* low-density lipoproteins, *VLDL* very low-density lipoproteins, *TP* total protein, *MDA* malondialdehyde, *NP-SH* nonprotein sulfhydryls. All values represent mean ± SEM. ^∗^*P* < 0.05; ^∗∗^*P* < 0.01; ^∗∗∗^*P* < 0.001; ANOVA, followed by Dunnett’s multiple comparison test. ^a^As compared with control group (GI); ^b^As compared with CCl_4_-only group (GII)

### Histological improvement by SSEE

**Fig. 2:**
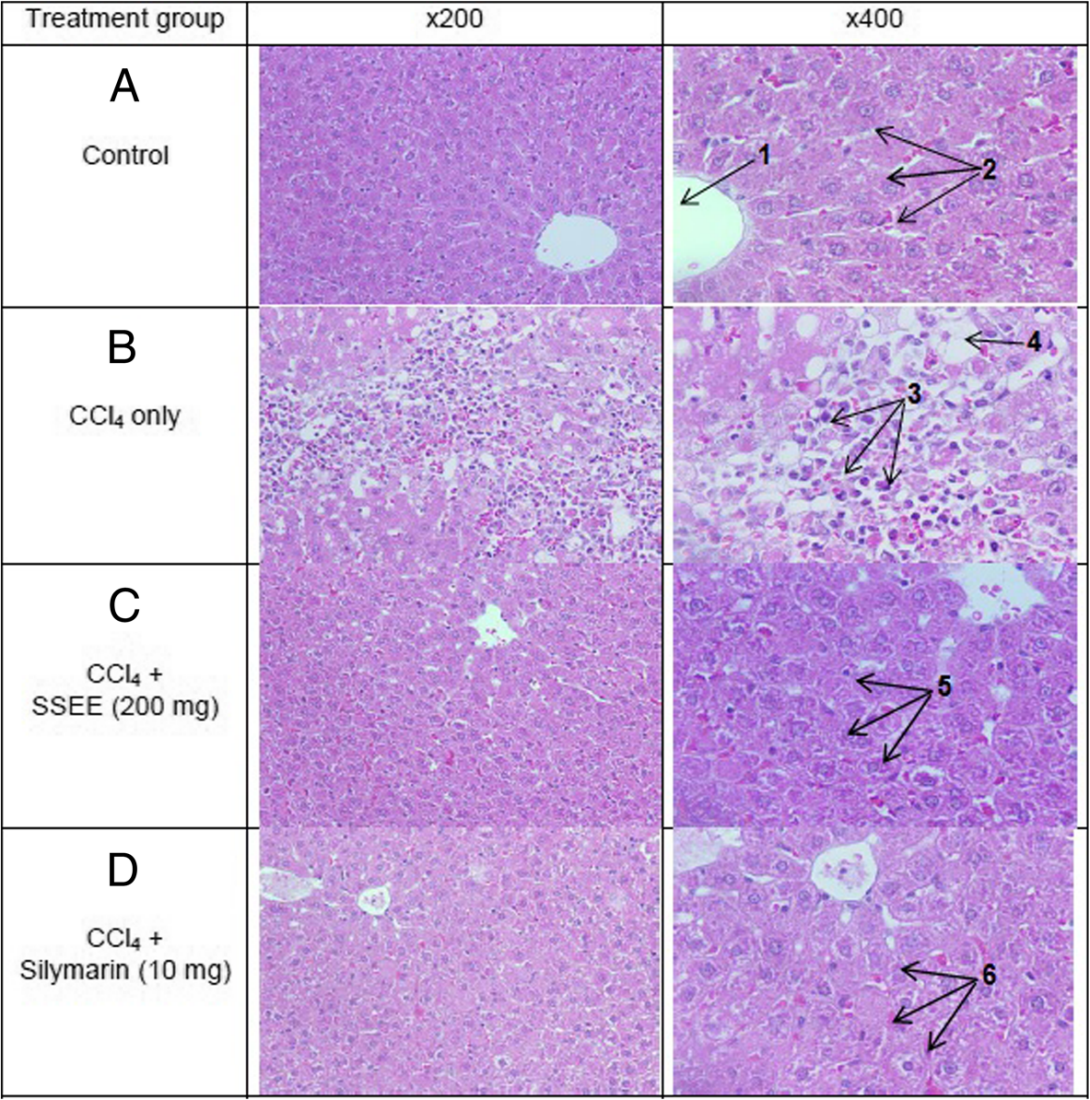
Histopathology of experimental rat liver. Histograms showing (**a**) healthy tissues with normal central vein and hepatocytes; (**b**) CCl_4_-injured tissue with necrosis and fatty degenerative changes; (**c**) tissue with normal hepatocytes and central vein with full recovery after CCl_4_ plus SSEE (200 mg.kg.bw) treatment; and (**d**) tissue with normal hepatocytes and fully recovered central vein after CCl_4_ plus silymarin (10 mg.kg.bw) treatment. 1. Central vein; 2. Normal hepatocytes cord; 3. Focal necrosis; 4. Vacuoles of fatty degeneration; 5. Normal hepatic tissue; 5. Normal hepatic tissue

The histopathological analysis showed congested central vein, necrosis, inflammatory cells and vacuoles of fatty degeneration in CCl_4_-treated rats liver (Fig. 2; panel b) as compared to the normal tissue of control group (Fig. 2; panel a). Treatment with the higher dose of SSEE (200 mg/kg.bw) (Fig. 2; panel c) or silymarin (10 mg/kg.bw) (Fig. 2; panel d) effectively attenuated the CCl_4_-induced toxicity, and significantly normalized hepatocytes lesion and resulted in full recovery.

### In vitro antioxidant activity of SSEE

**Fig. 3:**
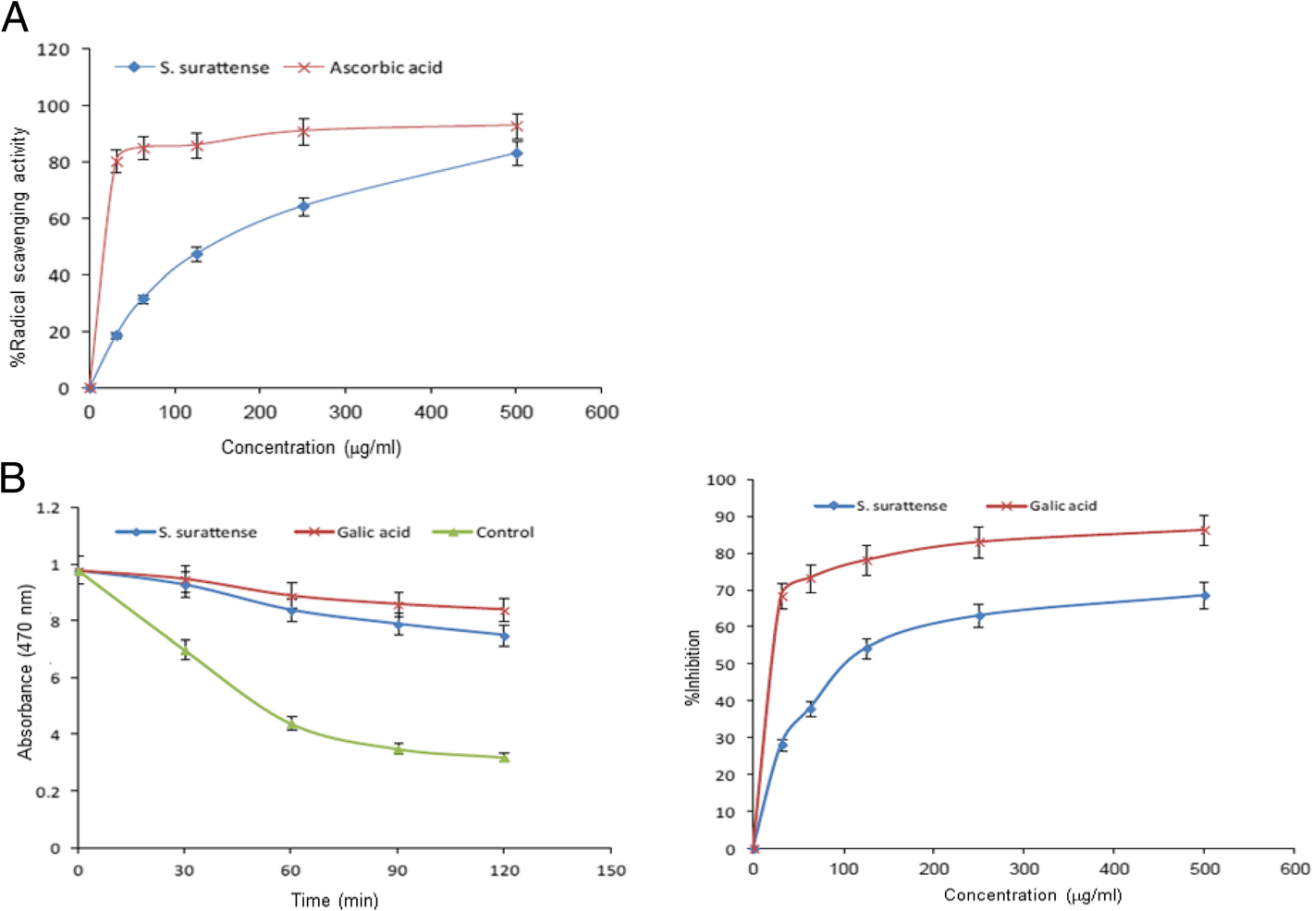
In vitro antioxidant activity of *S. surattense* ethanol-extract (SSEE). **a** DPPH radical scavenging activity of different concentrations (31.25–500 μg/mL) of total ethanol extract of *S. surattense* and standard antioxidant (Ascorbic acid). **b** β-carotene linoleic acid method showing β-carotene bleaching rate in presence of SSEE (500 μg/mL), gallic acid, blank control (left panel) and %inhibition of lipid peroxidation by different concentrations of SSEE (31.25–500 μg/mL) and gallic acid (right panel)

Radical scavenging activities of SSEE were found to be ~ 18.7, 31.6, 47.5, 64.5 and 83.3% at concentrations 31.25, 62.5, 125, 250 and 500 μg/mL, respectively, as compared to ascorbic acid (Fig. 3a). In line with this, SSEE also showed dose-dependent anti-lipid peroxidation activity compared to gallic acid (Fig. 3b).

### Phytochemical screening

Preliminary screening of SSEE indicated the presence of phytochemicals like, alkaloids, flavonoids, tannins, sterols and saponins but the absence of anthraquinones and cardiac glycosides (data not shown).

### Identification of β-sitosterol in SSEE

**Fig. 4:**
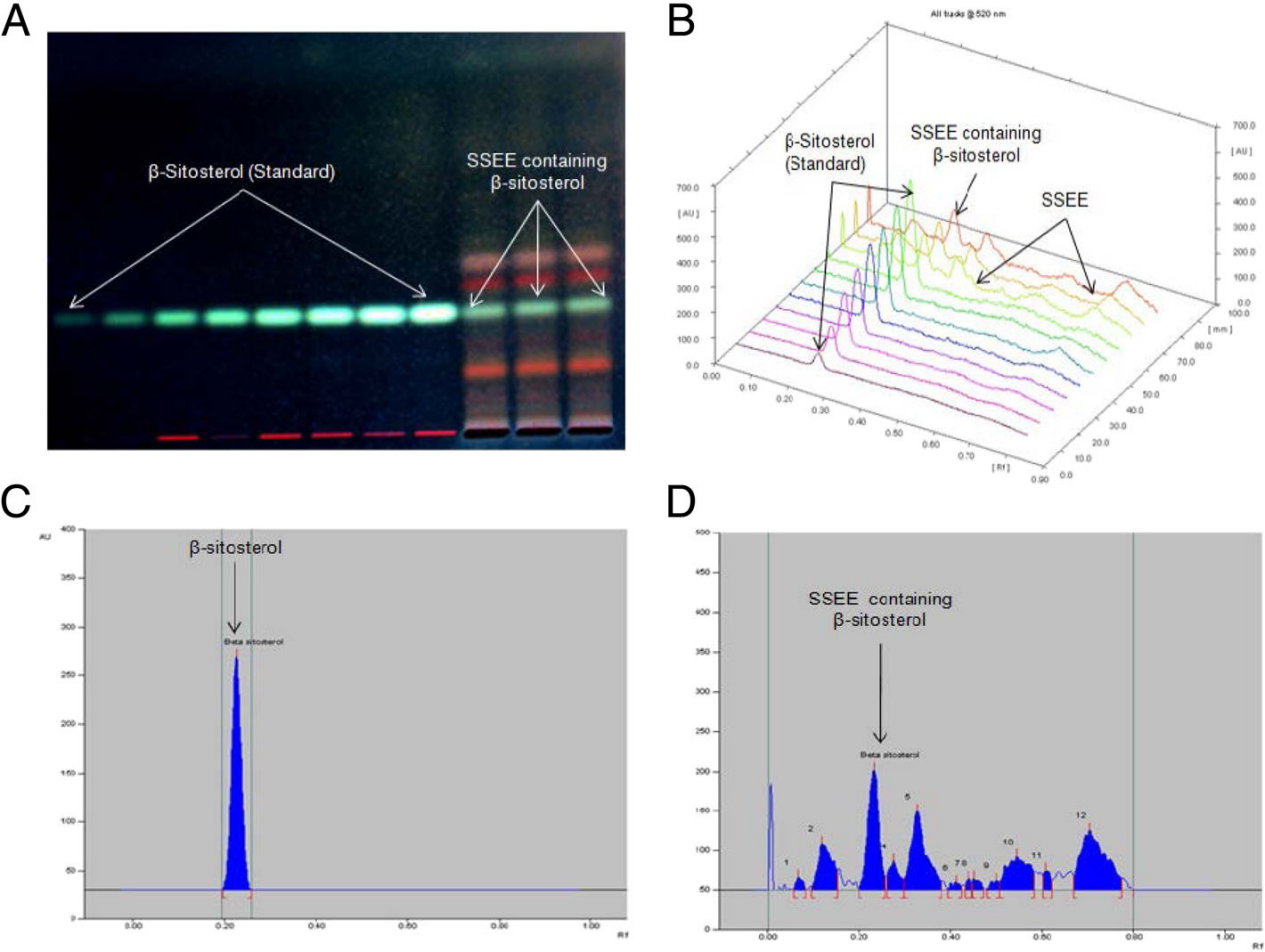
HPTLC standardization of β-sitosterol in the *S. surattense* ethanol-extract (SSEE, λ = 360 nm, mobile phase- hexane:ethyl acetate, 4:6, v/v). **a** chromatogram showing β-sitosterol biomarker (600 ng spot^− 1^, peak 1, R_f_ = 0.23); (**b**) chromatogram of SSEE containing β-sitosterol (peak 3, R_f_ = 0.23); (**c**) pictogram of developed and derivatized HPTLC plate of SSEE; (**d**) 3-D display of all tracks

Our HPTLC analysis identified β-sitosterol in SSEE as a compact spot at R_f_ = 0.23 (Fig. 4a, b), including good separations of different phytoconstituents (Fig. 4c, d). The estimated content of β-sitosterol was 3.46 μg/mg dry weight SSEE.

## Discussion

Over 35 medicinal plants are traditionally used in Saudi Arabia to treat various liver disorders where majority still lack scientific validations. To address this issue, we have recently demonstrated the in vitro and in vivo therapeutic potential of *A. mellifera* [9], *A. javanica* [10] and *A. subrecta* [11] against chemical-induced toxicity and oxidative damage, including growth stimulating activity of *S. surattense* on cultured HepG2 cells (unpublished data). To our knowledge, of the genus *Solanum*, the in vivo hepatoprotection by *S. nigrum* [29] and in vitro hepatoprotective as well as anti-oxidative effects of *S. melongena* [30] have been reported. The present study further investigated the healthprotective potential of genus *Solanum* and therefore, evaluated the anti-oxidative, anti-apototic and anti-hepatotoxic potential of *S. surattense* leaves ethanol extract (SSEE). Notably, the only in vitro study on *S. surattense* leaves extract has shown its anti-oxidative activity by DPPH free radical scavenging method [31].

In HepG2 cell culture model, we demonstrated the attenuation of DCFH-induced oxidative injury by SSEE in a dose-dependent manner. DCFH is generally used to measure in vitro oxidative stress generated by free radicals through the principle of oxidation of DCFH to the fluorescent DCF [32]. In addition, by exploiting DCFH as a potent cytotoxic agent in a panel of human cell lines, we have previously demonstrated protective salutations of several plant extracts or fractions against oxidative and apoptotic cell damages [9–11, 33, 34]. Moreover, by employing two different in vitro assays i.e., DPPH radical scavenging and linoleic acid bleaching methods, we showed the free radical attenuating and anti-lipid peroxidation potential of SSEE that further supported our data on HepG2 cells. Apoptotic cell death and inflammation by accumulation of endo/exogenous free radicals like reactive oxygen molecules are well known, where caspases (cysteine proteases) play essential roles [35]. Our anti-apoptotic signaling assay showed inhibition of DCFH-triggered cellular caspase-3 and -7 activations by SSEE in a dose-dependent manner that reversed HepG2 cell apoptosis.

Further, in vivo hepatoprotective activity of SSEE in CCl_4_-induced experimental hepatitis was evaluated in rats by examining elevated serum liver enzymes and cellular necrosis and membrane permeability [36]. Significant elevations in levels of serum ALT, AST, BIL, TC, TG, LDL and VLDL, and reduction in HDL (indicative of hepatic injury) were observed in CCl_4_-injured rats where treatment with SSEE (200 mg/kg.bw) or silymarin (10 mg/kg.bw) markedly normalized these parameters, showing healing and recovery of liver damage. Serum MDA is a marker of cell membrane damages due to lipid peroxidation [37] that was elevated in CCl_4_-treated rats. SSEE administration also normalized MDA, suggesting its in vivo hepatoprotective and curative effect. In addition, the diminished liver NP-SH level in CCl_4_-treated groups, a marker of oxidative hepatocellular damage [38] was replenished by SSEE or sliymarin, indicating amelioration of liver damage. Also, the decreased level of TP in hepatotoxic condition was normalized by SSEE or silymarin in CCl_4_-injured rats, confirming its therapeutic ability*.* Furthermore, the histopathological analysis of liver tissues revealed the presence of normal hepatic cords and absence of necrosis and lesser fatty infiltration in SSEE supplemented rats against CCl_4_-induced lesions.

Moreover, the qualitative phytochemical screening of SSEE showed presence of anti-oxidant polyhenols, sterols, flavonoids and saponins wherein HPTLC analysis further identified β-sitosterol that had been previously reported to protect the liver against CCl_4_-hepatotoxicity via enhancing mitochondrial glutathione redox cycling [39]. Taken together, our in vitro and in vivo results clearly and convincingly demonstrated the anti-oxidative and anti-apoptotic salutation of *S. surattense* against two different chemicals/toxins induced liver injury that was in agreement with our earlier findings [9–11].

## Conclusions

Our study of SSEE demonstrated its promising in vitro and in vivo anti-oxidative, anti-apoptotic and hepatoprotective potential against chemical-induced liver damage. This was further supported by phytochemical analysis as well as identification of β-sitosterol, a well-known anti-oxidant in SSEE. Probably the hepatoprotection by SSEE is due to multiple mechanisms involving the attenuation of the free radicals as well as apoptotic molecules, attributed to β-sitosterol. Our finding therefore, suggests therapeutic use *S. surattense* leaves as a source of dietary sterol in the prevention and treatment of chemical hepatitis and other chronic liver diseases. Nevertheless, further studies on novel active principles and their mechanism of action are warranted.

## Data Availability

The datasets used and analyzed during this study would be available upon request from the corresponding author.

## Abbreviations

ALP: Alkaline phosphatase
ALT: Alanine aminotransferase
AST: Aspartate aminotransferase
CCl_4_: Carbon tetrachloride
DCFH: 2,7-Dichlorofluorescein
DMSO: Dimethyl sulfoxide
DPPH: 1,1-diphenyl-2-picrylhydrazyl
HPTLC: High performance thin layer chromatographic
MDA: Malondialdehyde
MTT: 3-(4,5-dimethylthiazol-2-yl)-2,5-diphenyltetrazolium bromide
SSEE: *S. surattense* ethanol extract
TP: Total protein
